# Synthesis and Characterization of Dummy Template‐Based Molecularly Imprinted Polymers for Extraction of Aflatoxins in Food Samples

**DOI:** 10.1002/cbdv.202501749

**Published:** 2025-08-09

**Authors:** Mesha Mbisana, Dikabo Mogopodi, Moses T. Kabomo, Baagi T. Mmereki

**Affiliations:** ^1^ Department of Chemistry University of Botswana Gaborone Botswana

**Keywords:** adsorption studies, aflatoxins, dummy templates, food safety, molecularly imprinted polymers

## Abstract

MIPs have great potential for the selective adsorption of aflatoxins, due to their tailored binding sites. However, their synthesis using aflatoxins as templates is potentially hazardous and expensive. A viable solution to this challenge is to use dummy templates instead. Several dummy templates have been investigated for the synthesis of MIPs for aflatoxins. However, these studies have been conducted under varying conditions and by different research groups, resulting in a lack of systematic comparison. This makes it difficult to identify the optimal dummy template. In this study, five dummy templates and two functional monomers were evaluated to synthesize highly selective MIPs for aflatoxins. The polymers were produced through precipitation polymerization. FTIR, TGA, and SEM were employed to evaluate the structure, stability, and morphology of the material. The adsorption ability of the MIP and NIP was analyzed using LC–MS/MS. The findings revealed that MIPs synthesized using DMC exhibited the highest binding efficiency. In addition, MIPs created with functional monomers methacrylic acid (MAA) and methacrylamide (MAM) demonstrated similar adsorption abilities for aflatoxins. The adsorption process of the MIP is best described by the pseudo‐second‐order kinetic model and the Langmuir isotherm. The maximum monolayer adsorption capacities were measured at 8.13, 7.50, 8.38, and 7.63 mg/g for AFB_1_, AFB_2_, AFG_1_, and AFG_2_, respectively.

## Graphical abstract

Five dummy templates and two functional monomers were systematically evaluated for the preparation of selective MIPs for aflatoxins. 5,7‐Dimethoxycoumarin and methacrylic acid were found to produce highly selective MIPs, and the adsorbent was characterized by FTIR, TGA, and SEM. Absorption studies were carried out under varying conditions of contact time, initial aflatoxin concentration, pH, and temperature. The prepared MIP proved to be applicable to real‐world analysis of aflatoxins in cereal samples.

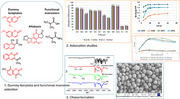

## 1 Introduction

Aflatoxins are toxic metabolites produced by *Aspergillus* fungi, posing significant health and economic challenges worldwide [1]. Chronic exposure to aflatoxins, often through contaminated food like grains and nuts, can lead to liver cancer, immune suppression, and stunted growth [2, 3]. Aflatoxins also contribute to significant economic losses in agriculture as contamination can lead to produce rejection for retail, lower market value, and trade limitations. In 2016, it was estimated that aflatoxins would cause the corn industry to lose between $52.1 million and $1.68 billion annually in the United States of America [4]. These losses are currently estimated to range from $17.5 million to $24.5 million per year on average [5], and researchers suggest that 89.5% of corn‐growing counties in 15 US states will experience increased aflatoxin contamination in 2031–2040 compared to 2011–2020 [6]. Developing countries are disproportionately affected due to inadequate food safety systems, exacerbating food insecurity. It is reported that annual losses due to aflatoxins in sub‐Saharan Africa amount to over $450 million in trade revenue of major staples, particularly maize and groundnuts [7]. Furthermore, mitigation costs, including public health initiatives, decontamination, and monitoring, further strain resources. The development of low‐cost and highly selective adsorbents, such as molecularly imprinted polymers (MIPs), enhances the efficiency and affordability of aflatoxin extraction and determination in food. This approach supports the Sustainable Development Goals (SDGs), particularly SDG 2 (Zero Hunger) and SDG 3 (Good Health and Well‐Being), by ensuring food security, food safety, and reduced exposure to toxic contaminants. It also aligns with SDG 12 (Responsible Consumption and Production) by minimizing the use of hazardous chemicals and promoting greener analytical methods.

Molecular imprinting technology (MIT) involves the development of MIPs with specific binding sites that are complementary in terms of functional groups, shape, and size to the template molecule. The polymers are achieved by co‐polymerizing functional and cross‐linking monomers in the presence of the template [8]. Subsequent removal of the template by solvent extraction exposes the complementary binding sites. Hence, MIPs can be used to rebind the target analyte with very high specificity [9, 10].

Precipitation polymerization is the simplest method for high‐yield synthesis of MIPs with uniform‐sized spherical particles and for large‐scale production [11]. Semong and Batlokwa [12] prepared an MIP for aflatoxin B_1_ (AFB_1_) using the precipitation method and obtained regular‐shaped spherical particles, resulting in high analyte recoveries (83.51%–90.03%).

**FIGURE 1:**
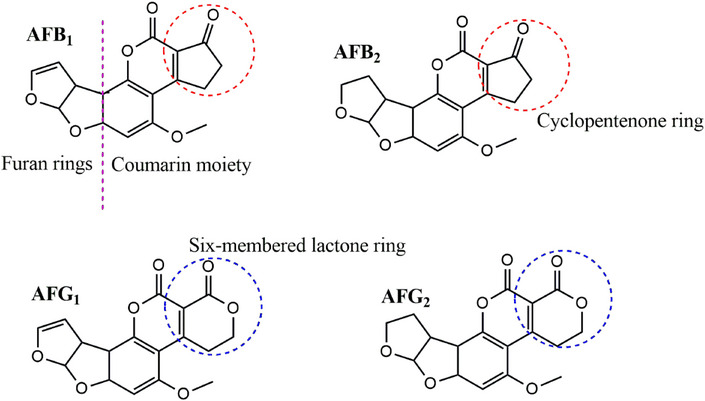
Chemical structures of AFB_1_, AFB_2_, AFG_1_, and AFG_2_. The red and blue circles indicate major differentiating structures between the B and G aflatoxins.

MIPs prepared using the target compounds as templates have limitations such as slow mass transfer and template leaching [13]. In addition, using aflatoxins as templates is expensive and potentially harmful to researchers. To overcome these challenges, “dummy” templates have been adopted to replace costly and toxic templates [14, 15]. Dummy, mimic, or fragment templates are structural analogs of the target analyte. They are similar to the target analyte in shape and functionality but are nontoxic and less expensive. Aflatoxins have a structure consisting of a coumarin moiety with a methoxy group and a difuran ring (Figure 1). The B and G aflatoxins are primarily differentiated by the ring fused to the coumarin structure, where the B aflatoxins have a cyclopentenone ring and the G aflatoxins have a six‐membered lactone ring fused to the coumarin structure. These are the major structural features that can be used to identify potential dummy templates for aflatoxins [16]. The computational modeling study by Wyszomirski and Prus [17] identified 5,7‐dimethoxycoumarin (DMC) as a structural analog for AFB_1_. This discovery has since prompted further research into the use of various coumarin derivatives for synthesizing MIPs for aflatoxins. Table 1 summarizes various MIPs developed for aflatoxins using AFB_1_ and dummy templates. Notably, coumarin derivatives have been widely explored due to their structural resemblance to the difuran ring of aflatoxins. For instance, MIPs prepared with 7‐methoxycoumarin [18] and 7‐acetoxy‐4‐methylcoumarin [19] demonstrated recoveries comparable to those of AFB_1_‐imprinted polymers. Thus, highlighting the effectiveness of these dummy templates. Beyond coumarins, other structurally diverse compounds such as 1‐hydroxy‐2‐naphthoic acid [20] and 6‐methyl‐4‐phenylchroman‐2‐one [21] have also yielded MIPs with excellent recognition properties, achieving recoveries between 65% and 96%. These findings underscore the versatility of the dummy imprinting strategy, which not only enhances safety but also reduces costs associated with handling highly regulated mycotoxins.

**TABLE 1:** MIPs prepared for aflatoxins using various templates.

Template	Application	Recovery (%)	Ref
AFB_1_	SPE in food and feed	83–96	[22]
AFB_1_	SPE on child weaning food	83.51–90.03	[12]
DMC	d‐SPE in fish feed	80–100	[23]
DMC	Magnetic sorbent for extraction in tea leaves and corn	75.6–94.8	[24]
DMC	Magnetic stir‐bar sorptive extraction (MSBSE) in baby food	39–60	[25]
7‐Methoxycoumarin	d‐SPE in various food matrices	79.1–109.4	[18]
7‐Ethoxycoumarin	SPE in peanuts	88–95	[26]
7‐Acetoxy‐4‐methylcoumarin	SPE in various food matrices	82.6–116.7	[19]
Ethyl 3‐coumarincarboxylate	Magnetic SPE in corn	75.1–99.4	[27]
6‐Methyl‐4‐phenylchroman‐2‐one	SPE in soy sauce	94–96	[21]
1‐Hydroxy‐2‐naphthoic acid	SPE in vegetables, fruits, and cereals	65–90	[20]

Although various dummy templates have been explored for molecular imprinting of aflatoxins, previous studies have employed different polymerization conditions (e.g., monomer ratios, cross‐linkers, and solvent systems). This diversity complicates the systematic comparison of the MIPs' performance. To address this limitation, 10 MIPs were prepared using 5 dummy templates and 2 functional monomers. This approach enables a direct evaluation of template‐monomer interactions and their impact on MIP selectivity for aflatoxins. To the best of our knowledge, this study is the first to systematically compare different dummy templates for MIP synthesis of aflatoxins. The prepared polymers were evaluated based on imprinting efficiency and adsorption efficiency for aflatoxins.

## 2 Results and Discussion

### 2.1 Template and Functional Monomer Selection

**FIGURE 2:**
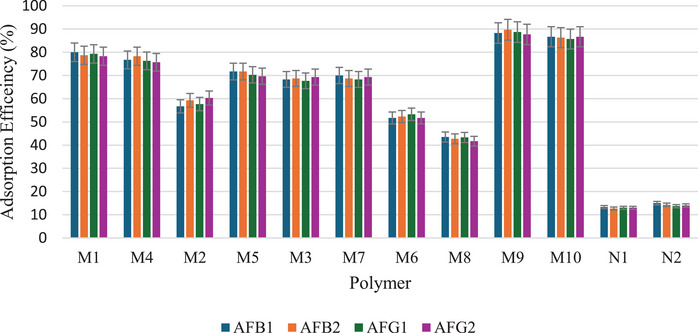
A bar chart illustrates the removal efficiency of MIPs (M1–M10) and NIPs (N1 and N2) for AFB_1_, AFB_2_, AFG_1_, and AFG_2_ (*n* = 3, %RSD > 5).

**FIGURE 3:**
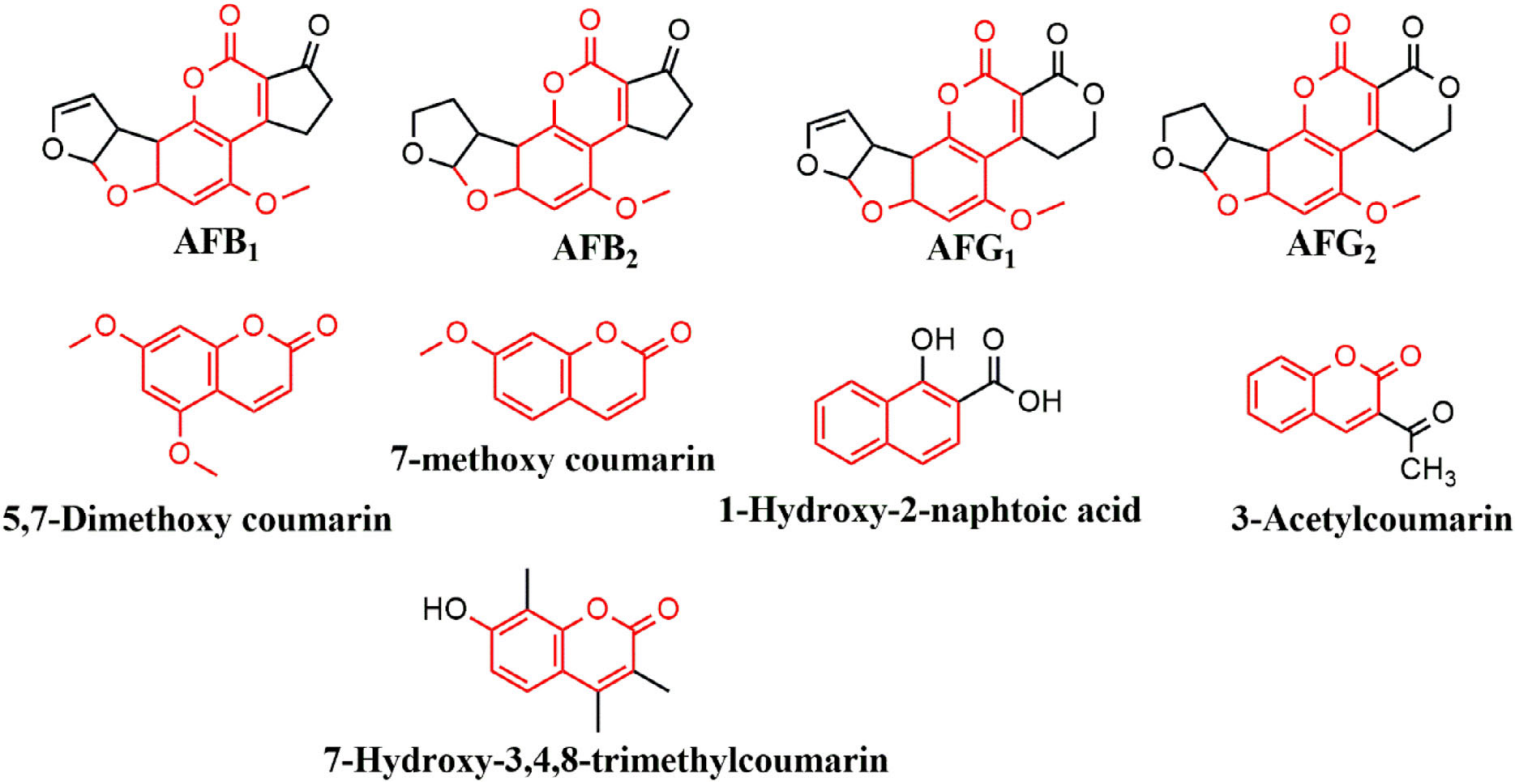
Structures of aflatoxins and dummy templates used in this study.

Five dummy templates and two functional monomers were investigated for their ability to form MIPs for aflatoxins. Table S1 shows the MIPs' performance parameters, such as removal efficiency (*R*%), adsorption capacity (*Q*_e_), and imprinting factor (IF) for individual analytes (using AFB_1_ as a test analyte). Figure 2 is a bar chart illustrating the removal efficiency of each polymer. Polymer M9 exhibited the highest adsorption efficiencies for all aflatoxins, ranging from 88.3% to 89.7%. In contrast, polymer M8 showed the lowest adsorption efficiencies, with values between 41.7% and 43.5%. These findings indicate that using DMC and methacrylic acid (MAA) in polymer M9 creates more complementary cavities in terms of functional groups and their arrangement, specifically tailored for the aflatoxins. MIPs imprinted by DMC have been reported to favor the extraction of aflatoxins [15, 23, 25]. The structure of DMC mirrors that of aflatoxins' coumarin moiety, including the positions of the methoxy groups at positions 5 and 7 (Figure 3). This structural similarity allows for the formation of complementary hydrogen bonding interactions between DMC and the functional monomers during polymerization, leading to well‐defined binding sites that enhance the selectivity and affinity of the MIPs for aflatoxins. Polymer M8 showed reduced adsorption efficiency for aflatoxins (41.7%–43.5%), most likely because specific binding sites between 3‐acetylcoumarin and methacrylamide (MAM) formed less effectively. Unlike DMC, 3‐acetylcoumarin lacks the ideal spatial arrangement and functional group orientation that closely mimics the coumarin moiety of aflatoxins. While it contains a carbonyl group at position 3, it does not offer strong or directional hydrogen bonding interactions with MAM's amide group, resulting in poorly defined or fewer selective binding sites in the resultant polymer. This consequently lowers the polymer's affinity and selectivity for aflatoxins throughout extraction. Thus, DMC and MAA were selected as the best combination for the MIP synthesis.

**FIGURE 4:**
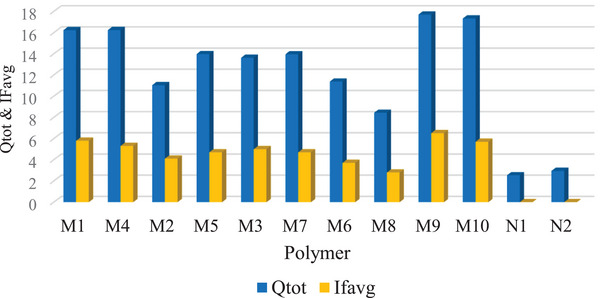
Comparison of adsorption capacities and imprinting factors of the prepared polymers.

Furthermore, M9 achieved the highest total adsorption capacity (*Q*_tot_) and average imprinting factor IF_avg_, measuring 17.7 mg/g and 6.5, respectively. In comparison, other MIPs had *Q*_tot_ and IF_avg_ values that ranged from 8.43 to 17.33 mg/g and 2.8 to 5.8, respectively (Figure 4). The non‐imprinted polymers (NIPs) had comparatively lower *Q*_tot_ values of 2.53 (N1) and 2.96 (N2). This shows that the high adsorption capacity in the MIPs is a result of the specific binding sites on the polymer created by the template.

**FIGURE 5:**
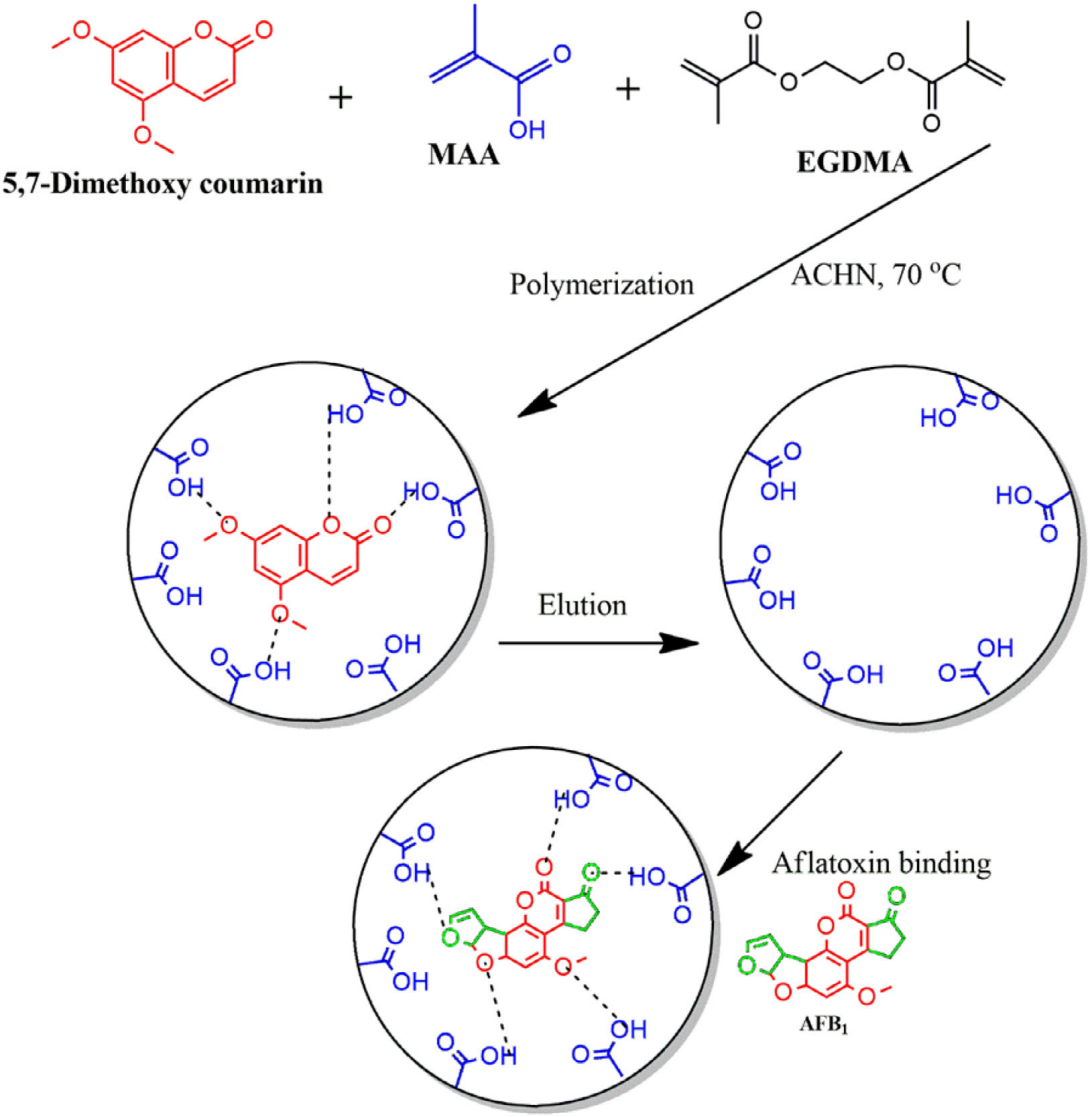
Diagram showing the synthesis of MIPs for aflatoxins using MAA and DMC.

The choice of functional monomer is critical in MIP synthesis, as it governs the strength and specificity of interactions between the monomer and the template during polymerization. Three MIPs prepared with MAA (M1, M6, and M9) demonstrated higher adsorption efficiencies than those prepared with MAM (M4, M8, and M10). However, in one instance, the MIP made with MAM (M5) exhibited a higher adsorption efficiency compared to the one made with MAA (M2). This was similar to the results obtained by Palmieri et al. [20], which showed that the combination of 1‐hydroxy‐2‐naphthoic acid and MAM produced a MIP with higher binding efficiency for aflatoxins. In the final pair, M3 (MAA) and M7 (MAM), the adsorption efficiencies were found to be similar. The differences in adsorption efficiencies between M9 and M10 samples were analyzed using a paired *t*‐test at a 95% confidence level to determine their significance (Equation 1).

(1) $$t_{\mathrm{statistic}}=\left|\bar{d}\right|\frac{\sqrt{n}}{S_{d}}$$

where *d* is the difference between the pair of results, *n* is the number of paired results, and S_d_ is the standard deviation of the differences, *d*. According to the test, *t*_stat_ was found to be lower than *t*_crit_ (0.0152 < 3.18). Thus, showing that there is no significant difference in adsorption efficiencies when using either MAA or MAM as the functional monomer with DMC as the template. Both MAA and MAM contain functional groups that can form non‐covalent interactions, particularly hydrogen bonds, with the functional groups in DMC (hydroxyl and carbonyl groups). The slightly improved performance of MIPs prepared with MAA may result from the stronger hydrogen bond donation capacity of the carboxylic acid group in MAA compared to the amide group in MAM. The carboxylic acid group in MAA facilitates robust interactions with the carbonyl and methoxy groups of DMC, potentially leading to better template recognition and stronger binding sites within the polymer matrix. Conversely, MAM's amide group provides more directional hydrogen bonding, which could enhance selectivity. However, in this case, the additional selectivity offered by MAM does not seem to significantly affect the overall imprinting efficiency or binding capacity compared to MAA. This finding suggests that the interactions provided by MAA are sufficiently strong and specific to form effective imprints for DMC. Hence, MAA was selected as the best functional monomer, and the interactions between MAA and DMC are illustrated in Figure 5. Moreover, the extensive body of literature supporting MAA's use in MIP synthesis further reinforces its suitability for this application [11, 18, 20, 28, 29, 30]. A recent study examined the effect of functional monomers on the binding properties of MIPs for selective recognition of dexamethasone (a corticosteroid used to reduce inflammation) [31]. Three monomers (MAA, *N*‐(hydroxymethyl)‐acrylamide, and 2‐hydroxyethyl methacrylate) were tested, and the results showed that MIPs prepared from 2‐hydroxyethyl methacrylate achieved the maximum analyte adsorption. The researchers concluded that this was due to the presence of more sites available for hydrogen bonding in 2‐hydroxyethyl methacrylate. These results also aligned with the predicted interaction strengths determined by computational simulations [31].

The MIPs' performance depends on both the dummy template and the chosen functional monomer. Notably, the same template can exhibit different performance outcomes depending on the functional monomer it interacts with. For instance, 1‐hydroxy‐2‐naphthoic acid combined with MAM outperformed its combination with MAA. In contrast, DMC exhibited similar performances when paired with either MAM or MAA. These findings suggest that hydrogen bonding plays a crucial role in MIP cavity formation and aflatoxin binding.

### 2.2 Characterization

#### 2.2.1 Fourier Transform Infrared

**FIGURE 6:**
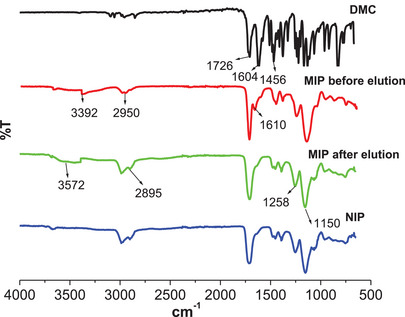
FTIR spectra of DMC, NIP, and M9 before and after DMC elution.

The Fourier transform infrared (FTIR) spectra of DMC, NIP, and M9 before and after DMC elution are shown in Figure 6. The peak observed at ≈1726 cm^−1^ in all spectra indicates C═O stretching vibrations, characteristic of the carbonyl groups present in DMC, MAA, and ethylene glycol dimethacrylate (EGDMA). The peak at ≈1604 cm^−1^ is ascribed to the C═C stretching in DMC, and its presence in MIP before DMC elution confirms that DMC is bound in the polymer network through hydrogen bonding with MAA. This peak is not observed in the MIP after elution and in the NIP. Thus, this confirms DMC removal in the polymer and the reversibility of the non‐covalent interactions (hydrogen bonds) between DMC and MAA. In addition, a broad peak at ≈3392 cm^−1^ ascribed to O─H stretching in MAA is observed in MIP before elution, and this peak broadens and increases in intensity after DMC elution at ≈3572 cm^−1^. This suggested that the carboxyl groups in MAA were exposed after DMC elution, leaving behind unbound O─H groups in the cavities. The sharp peaks at ≈1258 and 1150 cm^−1^ in DMC are ascribed to the C─O─C stretching in methoxy groups. These peaks are much broader and have higher intensity in the polymer spectra due to the additional C─O stretching in EGDMA. The peaks at ≈2985 and 1456 cm^−1^ are ascribed to the symmetric and asymmetric C─H stretching vibrations arising from the methyl and methylene groups of the polymer network. Both MIPs and NIPs have similar backbone structures; therefore, their spectra exhibit peaks at comparable positions, with only minor reductions in intensity observed in the NIP spectra due to the absence of template molecules. This has also been observed by other researchers [12, 18, 32].

#### 2.2.2 Scanning Electron Microscopy

**FIGURE 7:**
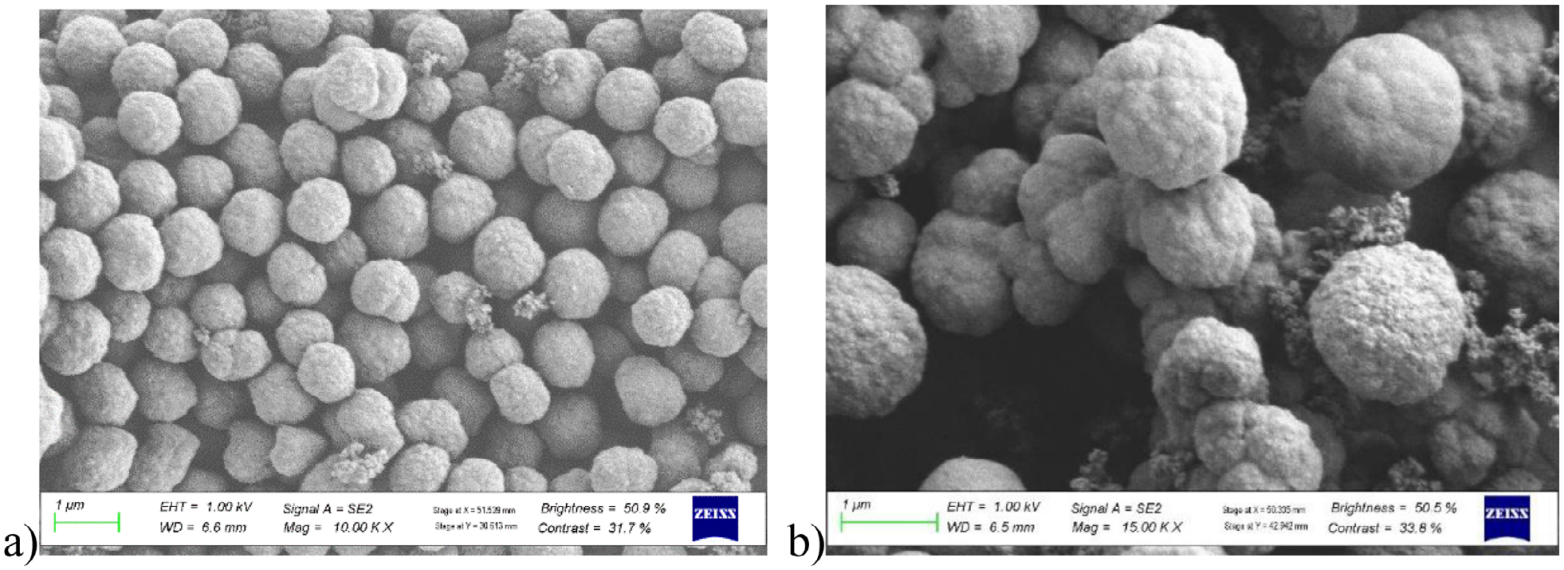
FESEM images of (a) M9 (MIP) and (b) N1 (NIP).

The surface morphology and particle size of M9 (MIP) and N1 (NIP) were examined using FESEM, as shown in Figure 7. Before imaging, the samples were mounted on aluminum stubs with double‐sided carbon tape. The samples were then coated with a thin (≈10 nm thick) layer of gold, using a Leica EM ACE200 Gold Sputter Coater. This is done to make the sample surface electrically conductive to avoid electron build‐up on the sample surface that can cause electron charge. The MIP and NIP morphologies are similar, with regular spherical‐shaped microparticles (diameter ≈ 1 µm). This particle shape is commonly obtained in precipitation polymerized polymers [10]. The rough surface of the MIP is influenced by the presence of cavities formed after template removal. In addition, the particles are well‐dispersed, which is conducive to the rapid binding of targeted molecules. Furthermore, the small particles are ideal for adsorption since they generally have a larger surface area. This is important for aflatoxin preconcentration and accurate subsequent quantification. Similar aflatoxin MIP particles have been achieved by other researchers through precipitation polymerization. For example, Semong and Batlokwa [12] obtained regular spherical shapes and sizes (diameter ≈ 0.8 µm).

#### 2.2.3 Thermogravimetric Analysis

**FIGURE 8:**
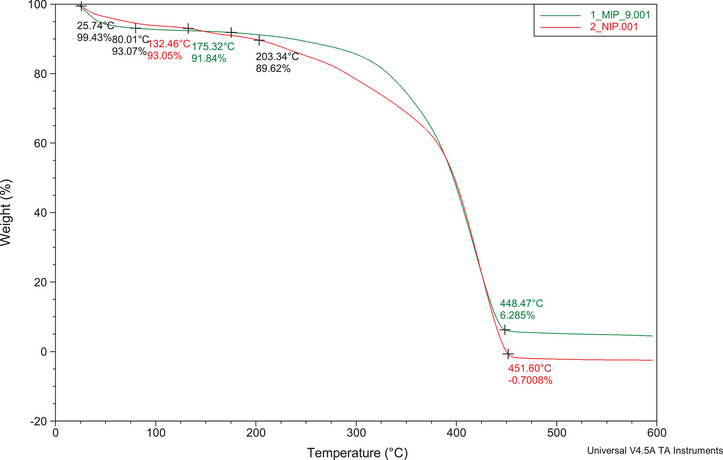
TGA curve for decomposition of M9 and N1.

Thermogravimetric analysis (TGA) enables the assessment of the decomposition characteristics of the synthesized polymers. The TGA plots obtained (25°C–600°C, nitrogen gas) for the MIP and NIP showed similar characteristics (Figure 8). The first stage of weight loss (∼6%) was observed between 25.47°C and 80.01°C, which is mainly due to moisture loss. Thereafter, the MIP weight loss remained constant from 80.01°C to 175.32°C and drastically reduced by ∼86% till 448.47°C (second stage of weight loss). This was due to the degradation of the main polymer backbone. Finally, the MIP curve remained constant until 600°C. This is attributed to the MIPs' thermal resistance, whereby the amount of polymer left was ∼6%. On the other hand, the NIP weight loss remained constant from 80.01°C to 132.46°C and drastically reduced to 0% till 451.60°C. This data shows that polymerization in the MIP results in more thermally stable adsorbents than in the NIP due to stabilization by the template molecules. These results align with the scanning electron microscopy (SEM) images that suggested uncontrolled morphology in the NIPs.

### 2.3 Adsorption Studies

#### 2.3.1 Effect of Initial Concentration

**FIGURE 9:**
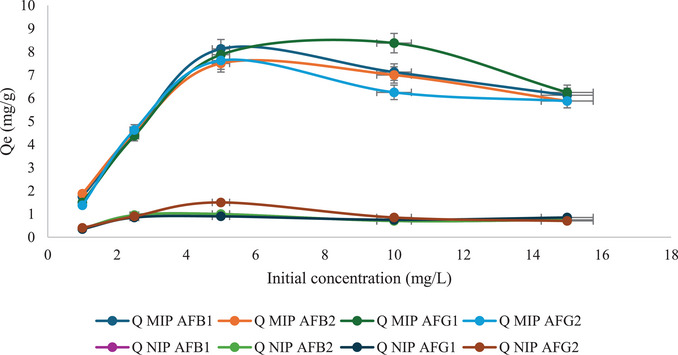
Adsorption capacities of M9 and NIP for the adsorption of aflatoxins at different initial concentrations (*n* = 3, %RSD < 10%).

Figure 9 shows the effect of initial aflatoxin concentration (1–15 mg/L) on the total adsorption capacity of the M9 and N1. The adsorption capacity of M9 toward AFB_1_ and AFB_2_ increased gradually from 1 to 5 mg/L and then gradually decreased until 15 mg/L. Whereas, for AFG_1_ and AFG_2_, the adsorption capacity increased from 1 to 10 mg/L and then decreased to 15 mg/L. This shows that as the concentration increased, M9 reached points of saturation for the aflatoxins at 5 and 10 mg/L. The total adsorption capacity of M9 at 5 and 10 mg/L was calculated as 31.13 and 28.75 mg/g, respectively. Thus, the initial concentration of mg/L was determined to be the point of equilibrium for total aflatoxin adsorption. At higher concentrations than this point, M9 was saturated with the aflatoxins, and there were no available specific binding sites for aflatoxin adsorption. Hence, a decrease in the adsorption capacity was observed. This phenomenon has been observed in other studies; for example, Wei et al. [22] reported that the binding capacity of MIP increased with increasing AFB_1_ concentration until it reached an equilibrium state. M9 exhibited much higher adsorption capacity compared to the NIP because of the presence of specific binding sites created by DMC, which are complementary to aflatoxins in terms of shape and functional groups. In contrast, the NIP does not have these specific binding sites, hence lower adsorption capacity.

The data obtained was fitted to the nonlinear Langmuir model to evaluate the binding energy (equilibrium constant) and number of available binding sites (maximum binding capacity) (Figures S1–S4). Table 2 shows the Langmuir constants and the corresponding correlation coefficients (*R* ^2^) for the adsorption of each analyte. This model assumes that adsorption happens on a monolayer surface with a finite number of adsorption sites and uniform adsorption energies [33]. This is expected in MIPs since they have specific cavities engineered to bind target analytes. The theoretical maximum adsorption capacities (*Q*_max_) for AFB_1_, AFB_2_, AFG_1_, and AFG_2_ are 7.07, 6.80, 10.43, and 6.52 mg/g. The experimental *Q*_max_ values were 8.13, 7.50, 8.38, and 7.63 mg/g. The dimensionless Langmuir equilibrium parameters, *R*_L_ values at different initial concentrations, are listed in Table 3. The values all fall within 0 < *R*_L_ < 1, which means that the adsorption of aflatoxins at all concentrations is favorable.

**TABLE 2:** Langmuir isotherm constants and *R* ^2^ values.

Aflatoxin	Langmuir constants		
	*Q*_max_ (mg/g)	*K*_L_ (L/g)	*R* ^2^
B_1_	7.07	1.77	0.9046
B_2_	6.80	2.14	0.9135
G_1_	10.43	0.92	0.8791
G_2_	6.52	1.60	0.7492

**TABLE 3:** Langmuir isotherm *R*_L_ values at different initial concentrations.

*R*_L_ values				
*C*_o_ (mg/L)	AFB_1_	AFB_2_	AFG_1_	AFG_2_
1.0	0.361	0.318	0.521	0.385
2.5	0.184	0.157	0.303	0.200
5.0	0.102	0.085	0.179	0.111
10.0	0.053	0.045	0.098	0.059
15.0	0.036	0.030	0.068	0.040

#### 2.3.2 Effect of Contact Time

The influence of contact time on the adsorption capacity of M9 and N1 is shown in Figures S5 and S6. The adsorption capacity increased with the contact time, reaching an equilibrium state at 15 min, after which it remained constant. The highest total adsorption capacity observed was 29.38 mg/g. The adsorption behavior between the adsorbent and the adsorbates was described using kinetic parameters derived from the intraparticle diffusion, pseudo‐first‐order, and pseudo‐second‐order models. The intraparticle diffusion kinetic model describes the rate of adsorbate diffusion during the adsorption process [11, 34]. Whereas, the pseudo‐first‐order model is used to describe physical adsorption of adsorbates from aqueous solution onto solid adsorbents and assumes that the rate‐limiting step is solute adsorption onto the adsorbent [35]. The pseudo‐second order model indicates that the adsorption of adsorbate onto the surface of adsorbents occurs through a chemisorption mechanism and that the adsorption mechanism might depend on both the adsorbates and the adsorbents [28].

**FIGURE 10:**
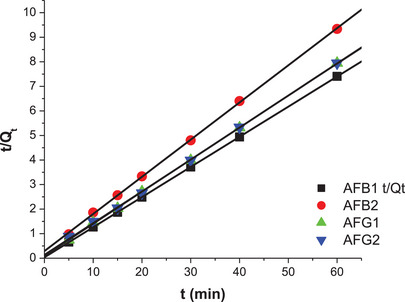
Pseudo‐second‐order kinetic model linear plots for adsorption of aflatoxins by M9.

**FIGURE 11:**
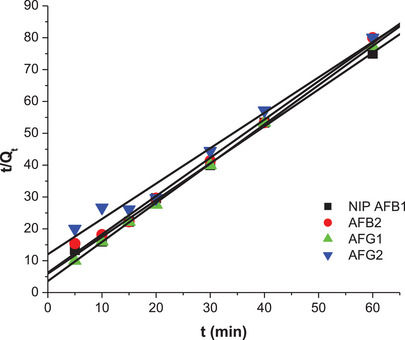
Pseudo‐second‐order kinetic model linear plots for adsorption of aflatoxins by N1.

Figures 10 and 11 and Table 4 show the linearized plots and constants for the pseudo‐second‐order model for both M9 and N1. The data show that *R* ^2^ values for the pseudo‐second model were closer to 1 for the aflatoxins. Hence, this model well describes the adsorption of aflatoxins onto M9 and N1. This means that chemical adsorption primarily affected the adsorption rates of the polymers. The adsorption capacities of M9 (*Q*_e_) were 8.15, 6.61, 7.67, and 7.74 mg/g for AFB_1_, AFB_2_, AFG_1_, and AFG_2_, respectively. These adsorption capacities were higher than those for the NIP, which were 0.86, 0.83, 0.81, and 0.90 mg/g, respectively. This is because M9 has specific binding sites for aflatoxins, whereas NIP lacks these tailored binding sites, hence its poor adsorption of the aflatoxins.

**TABLE 4:** Linear equations and constants for the pseudo‐second‐order kinetic model for both M9 and NIP.

	Aflatoxin	Linear equation	*R* ^2^	*Q*_e_ (mg/g) {1/slope}	*K*_2_ (mg/g/min) {slope^2^/intercept}
M9 (MIP)	B_1_	*Y* = 0.12275*x* + 0.03506	1	8.15	0.0430
	B_2_	*Y* = 0.15135*x* + 0.28987	0.9999	6.61	0.0790
	G_1_	*Y* = 0.13046*x* + 0.10266	0.9999	7.67	0.1658
	G_2_	*Y* = 0.12915*x* + 0.15649	0.9997	7.74	0.1066
N1 (NIP)	B_1_	*Y* = 1.15679*x* + 5.89937	0.9986	0.86	0.2268
	B_2_	*Y* = 1.20206*x* + 6.25135	0.9971	0.83	0.2311
	G_1_	*Y* = 1.23047*x* + 3.58227	0.9999	0.81	0.4227
	G_2_	*Y* = 1.11078*x* + 12.00439	0.9911	0.90	0.1028

Figures S7 and S8 show the linear plots for the pseudo‐first‐order model for M9 and N1, respectively, whereas Table S2 shows the equations of the lines and pseudo‐first‐order model constants for the adsorption of aflatoxins. The *R* ^2^ values ranged from 0.78278 to 0.91301, which is lower than those for the pseudo‐second‐order model. The linear plots and constants of the intraparticle diffusion model are shown in Figure S9 and Table S3, respectively. The *R* ^2^ values from these plots were also much lower than 1 (0.6632–0.8770), which means that interparticle diffusion did not occur during the adsorption process. In addition, the linear plots did not pass through origin, which meant that intraparticle diffusion was not the main process occurring during the adsorption.

#### 2.3.3 Effect of pH

**FIGURE 12:**
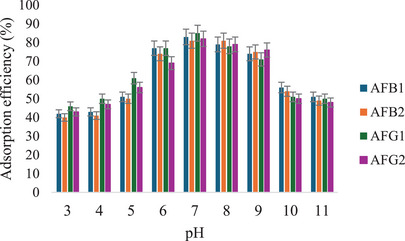
The effect of pH on the adsorption efficiency of M9 for aflatoxins (*n* = 3, %RSD > 10%).

The effect of pH (3–11) on the removal of aflatoxins by the MIP was studied, and the results are shown in Figure 12. These results show that the removal of aflatoxins by M9 is influenced by pH. The highest adsorption efficiencies (81%–85%) were observed at neutral pH, while much lower efficiencies were recorded at higher and lower pH levels. This may be due to the protonation and deprotonation of the aflatoxins at low and high pH conditions, respectively [36]. At low pH, the carboxyl groups in MAA are protonated, thus reducing their ability to interact with the aflatoxins through hydrogen bonding [11]. On the other hand, aflatoxins' hydroxyl groups are deprotonated at high pH, leading to electrostatic repulsion of the negatively charged polymer surface.

#### 2.3.4 Adsorption Thermodynamics

The Van't Hoff plots for the adsorption of aflatoxins at 298, 318, and 338 K are shown in Figure S10, and the *R* ^2^, Δ*H*°, Δ*S*°, and Δ*G*° are shown in Table 5. All Δ*H*°, Δ*S*°, and Δ*G*° values were negative, indicating that the adsorption of aflatoxins onto the MIP is exothermic and spontaneous. The highest negative Δ*G*° (−5.37 to −5.65 kJ/mol) values were observed at room temperature (298 K), where no energy input is required. As the temperature increased, Δ*G*° became less negative, indicating that the adsorption of aflatoxins onto the MIP surface is less favorable at high temperatures. This was expected, since sorption is an exothermic process [37].

**TABLE 5:** Thermodynamic parameters for the adsorption of aflatoxins by M9.

Aflatoxin	Equation of the line	*R* ^2^	Enthalpy, Δ*H*° (kJ/mol)	Entropy, Δ*S*° (J/K mol)	Gibbs energy, Δ*G*° (kJ/mol)		
					298 K	318 K	338 K
AFB_1_	*Y* = 2047.5*x* − 4.6305	0.9656	−17.02	−38.50	−5.65	−4.55	−4.13
AFB_2_	*Y* = 2279.6*x* − 5.448	0.999	−18.54	−45.29	−5.47	−4.50	−3.67
AFG_1_	*Y* = 2025.7*x* − 4.583	0.9979	−16.84	−38.10	−5.59	−4.76	−4.09
AFG_2_	*Y* = 1548.1*x* − 3.0281	0.9902	−12.87	−25.18	−5.37	−4.73	−4.37

### 2.4 MIP Selectivity (Competitive Adsorption)

The selectivity of M9 and N1 toward aflatoxins was investigated by conducting a removal process experiment using three different mycotoxins (fumonisin B_1_ [FB_1_], ochratoxin A [OTA], and zearalenone [ZEA]) that usually co‐occur with aflatoxins. Table 6 shows the removal percentages of all selected compounds by M9 and NIP. The removal percentage for aflatoxins ranged from 70.0% to 72.6%, and 8.8% to 11.4% by M9 and N1, respectively. These results further prove that the MIP has specific binding sites for aflatoxins while the NIP does not. The removal percentage for competing mycotoxins ranged from 3.2% to 22.0% for both M9 and N1. These lower percentages indicate that the competing mycotoxins had minimal binding on M9 and N1 surfaces.

**TABLE 6:** Competitive adsorption of aflatoxins, FB1, OTA, and ZEA by MIP and NIP.

Compound	*R* (%) MIP	*R* (%) NIP	*K*_D_ MIP (mg/g)	*K*_D_ NIP (mg/g)	*K* MIP	*K* NIP	*K*′
AFB_1_	71.0	11.4	8.875	1.425	3.23	3.56	0.906
AFB_2_	72.6	10.0	9.075	1.25	3.30	3.13	1.056
AFG_1_	70.4	11.0	8.8	1.375	3.20	3.44	0.931
AFG_2_	70.0	8.8	8.75	1.1	3.18	2.75	1.157
FB_1_	10.0	10.8	1.25	1.35	—	—	—
OTA	22.0	3.2	2.75	0.4	—	—	—
ZEA	15.0	8.6	1.875	1.075	—	—	—

In addition, M9 selectivity was evaluated using the distribution coefficient, *K*_D_. High *K*_D_ values mean the adsorbent is highly selective toward target analytes [11]. The *K*_D_ values obtained for the adsorption of aflatoxins by M9 ranged from 8.80 to 9.08 mg/g, while those for the NIP ranged from 1.10 to 1.43 mg/g. These results show that the MIP is more selective than the NIP. Furthermore, the selectivity of M9 for aflatoxins in the presence of competitors/interferents was confirmed using the selectivity coefficient, *K*. *K* > 1 means the MIP binds targeted analytes more selectively than competing compounds, and the higher the *K* value, the stronger the imprinting effect. Whereas *K* ≈ 1 or *K* < 1 means the MIP has poor selectivity. *K* values for M9 ranged from 3.18 to 3.30, which indicates strong selectivity toward aflatoxins in the presence of competing mycotoxins. Finally, the relative selectivity, *K*′ of M9, was examined as a ratio of *K*_D_ MIP over *K*_D_ NIP. *K*′ values that ranged from 0.906 to 1.157 were observed, which confirmed that M9 had a greater affinity for aflatoxins than the NIP.

### 2.5 MIP Reusability in Real Food Samples

**FIGURE 13:**
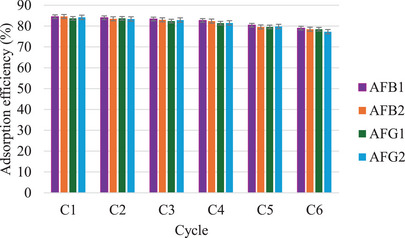
Reusability study of M9 in maize samples.

To investigate the reusability of the MIP sorbent, the adsorption‐desorption cycle was studied six times using the same MIP sorbent to extract spiked aflatoxins from blank maize samples. The extraction was carried out according to a dispersive solid phase extraction (d‐SPE) method reported by Thati et al. [18]. About 2 g each of homogenized maize samples was spiked with 5 mg/L aflatoxin, then extracted with 10 mL MeCN/H_2_O (75:25, v/v). The samples were vortexed for 2 min and sonicated for 5 min for maximum extraction. Thereafter, the extract was centrifuged at 6000 rpm, and the supernatant was collected into 15 mL centrifuge tubes. The MIP (4 mg) was dispersed into the supernatant and vortexed for 15 min, after which the adsorbent was retrieved by centrifugation, washed with MeOH/AcOH (98:2 v/v), and then reused. Figure 13 shows that the adsorption efficiencies ranged from 81.5% to 84.5% for the first four cycles. After that, lower adsorption efficiencies ranging from 77.3% to 79.8% were observed from the last two cycles. The decrease in adsorption efficiency is due to rewashing and destruction of some of the cavities on the surface of MIP [37]. Overall, the MIP showed good stability and reusability, which indicates it may be an economically feasible adsorbent for the removal of aflatoxins from food. On top of that, the high recoveries show the MIP is applicable to real food samples.

### 2.6 Comparison With Other MIPs

Table 7 shows a comparison of the maximum adsorption capacities and selectivity coefficients for M9 with previously reported MIPs for aflatoxins. Although a majority of previous studies did not investigate the adsorbent's adsorption mechanisms and selectivity coefficients, valuable insights into how template selection and functional monomers influence polymer performance can be derived. MIPs utilizing MAA as the functional monomer demonstrated superior adsorption capacities, particularly when imprinted with AFB_1_ or its structural analog, DMC. The AFB_1_‐imprinted MIP reported by Pezeshkpur et al. [38] exhibited the highest maximum adsorption capacity (8.975 mg/g), closely followed by the DMC‐imprinted MIP in this study (7.50–8.38 mg/g). This suggests that MAA's carboxylic acid groups facilitate strong non‐covalent interactions (hydrogen bonding and electrostatic forces) with aflatoxin molecules, enhancing binding affinity. In contrast, MIPs using MAM or alternative templates exhibited significantly lower adsorption, likely due to weaker molecular recognition. Thus, reinforcing the importance of both monomer choice and template complementarity in MIP design.

**TABLE 7:** Comparison of the maximum adsorption capacities and selectivity coefficients for M9 with previously reported MIPs for aflatoxins.

Template used	Functional monomer	*Q*_max_	*K*_D_	*K*	*K*′	Ref
DMC	MAA	7.50–8.38 mg/g	8.8–9.075 mg/g	3.18–3.30	0.906–1.157	This study
AFB_1_	MAA	8.975 mg/g	0.231–2.546	7.316–11.022	0.199–0.506	[38]
1‐Hydroxy‐2‐naphthoic acid	MAM	48.21–50.46 ng/mg	—	—	—	[20]
7‐Acetoxy‐4‐methylcoumarin	MAA	3.0 µg/mg	—	—	—	[19]
6‐Methyl‐4‐phenylchroman‐2‐one	MAA	—	—	—	—	[21]
DMC	MAA and 4‐vinylpyridine	—	—	—	—	[24]
AFB_1_	MAA	8.2 mg/g	51.6 mL/g	1.98	1.6	[22]

Selectivity analysis further revealed key differences in molecular recognition capabilities. While AFB_1_‐imprinted MIPs displayed the highest selectivity (7.316–11.022), their relative selectivity coefficients (0.199–0.506) were comparatively low, indicating strong specificity for AFB_1_ but limited recognition of related mycotoxins. In contrast, DMC‐imprinted MIP in this study exhibited moderate selectivity (3.18–3.30) but higher relative selectivity (*K*′ ≈ 1), making it more versatile for applications requiring broad‐spectrum aflatoxin detection. Earlier studies, such as Wei et al. [22] reported comparable adsorption capacities (8.2 mg/g) but lower selectivity (*K* = 1.98), likely due to differences in polymerization conditions or template‐monomer stoichiometry. Overall, M9 had comparable adsorption capacities to other reported MIPs, thus showing that it is a promising material for the removal of aflatoxins.

## 3 Experimental

### 3.1 Chemicals and Reagents

Mycotoxin standards (AFB_1_, AFB_2_, AFG_1_, AFG_2_, FB_1_, OTA, and ZEA) were purchased from the National Metrology Institute of South Africa (NMISA, Pretoria). MAA, MAM, DMC, 7‐methoxycoumarin, 1‐hydroxy‐2‐naphthoic acid, 7‐hydroxy‐3,4,8‐trimethylcoumarin, 3‐acetlycoumarin, 1,1′‐azobis(cyclohexanecarbonitrile) (ACHN), EGDMA, and toluene were purchased from Sigma‐Aldrich (Germany). LC–MS grade solvents for chromatography, ultrapure water (H_2_O), methanol (MeOH), and acetonitrile (MeCN) were purchased from Merck, Supelco (Germany). Formic acid (FA) (ACS, >98%) was purchased from Carl Roth (Germany).

### 3.2 Equipment

Sonication was performed on a Biopeak digital ultrasonic bath, voltage 220 V/50 Hz. The Polymers were characterized by a PerkinElmer. Precisely, the Spectrum 100 FTIR spectrometer. TGA was conducted on a TGA Q500 V6.7 Build 203 instrument, from 25°C to 600°C, under nitrogen gas. SEM analysis was carried out in a Zeiss MERLIN Field Emission Scanning Electron Microscope (Carl Zeiss Microscopy, Germany), beam condition 1 kV, 100 IProbe. Chromatographic measurements were performed using an AB Sciex Instruments ExionLC LC coupled with a linear ion trap quadrupole (QTRAP 6500+) mass spectrometer. The instrument was equipped with a binary gradient AD pump, AD autosampler, AD column oven quaternary high‐pressure pump, and an electrospray ionization (ESI) (IonDrive TM Turbo V source). A Utilsil Plus C18 analytical column, 5 µm, 2.1 × 150 mm (Welch, WV, USA) was used.

### 3.3 Selection of a Dummy Template and Functional Monomer

The approach was to investigate and select a dummy template and functional monomer that could be used to synthesize an MIP with high adsorption efficiency for aflatoxins. Five dummy templates were chosen based on their structural orientation (Figure 3). Four dummy templates with a coumarin moiety: DMC, 7‐methoxy coumarin, 3‐acetyl coumarin, 7‐hydroxy‐3,4,8‐trimethyl coumarin; and one template without the coumarin moiety 1‐hydroxy‐2‐naphthoic acid. 1‐Hydroxy‐2‐naphthoic acid was selected due to its low cost and its reported ability to effectively mimic all four aflatoxins, as it lacks the oxygen intercalated in the aromatic rings [20]. Two functional monomers, MAA and MAM, were chosen based on the established ability to form stable complexes with templates [20, 39]. In all the studies, EGDMA served as a cross‐linker due to its high cross‐linking efficiency from the two methacrylate groups. These groups ensure the formation of stable and selective binding sites. Its excellent solubility in porogens, chemical stability, and compatibility with various functional monomers make it a versatile and cost‐effective choice [40]. There were 10 MIPs synthesized, and all were screened as potential aflatoxin adsorbents.

### 3.4 MIP Synthesis

A mole ratio of 1:4:20 for the template: functional monomer: cross‐linker was used to synthesize both MIPs and NIPs. All the polymers were synthesized using an adopted precipitation polymerization method with modifications [23]. Table S1 shows the polymers prepared with different templates and functional monomers. A mass of 0.1 g (≈ 0.6 mmol) of the template and 2000 µL of MAA or 0.2 g MA (≈ 2.4 mmol) were added to a 50 mL reaction bottle containing 40 mL of MeCN/toluene (1:3 v/v) solution. The mixture was left to rest at room temperature for 1 h to allow for template‐functional monomer self‐assembly. Then, 2000 µL (12.0 mmol) EGDMA and 0.4 g ACHN were added to the reaction bottle. The mixture was sonicated for 10 min and then degassed with nitrogen for 10 min. Thereafter, the mixture was sonicated for 1 h in a hot water bath at 70°C. Then, polymerization was allowed to complete for a further 5 h in the hot water bath without sonication. The prepared MIP particles were washed with MeOH/AcOH (9:1 v/v) in a Soxhlet extractor. Complete template removal was confirmed by LC–MS/MS. The NIPs were prepared in the same way, but without the addition of the template.

### 3.5 MIP Screening

A 10 mL solution of MeOH/MeCN (50:50 v/v) was spiked at 2 mg/L of each aflatoxin. A mass of 4 mg of the polymer was then added to the solution and allowed to adsorb aflatoxins for 1 h over a rotary shaker. Thereafter, the mixture was centrifuged for 10 min. The supernatant was then collected, dried over a gentle stream of nitrogen gas, and reconstituted with 1 mL MeOH/H_2_O (50:50 v/v), and filtered through 0.45 mm frits. The filtrate was analyzed for aflatoxins using an LC–MS/MS. The same procedure was done simultaneously for all the synthesized polymers. An aflatoxin that was favored by a specific MIP showed a low peak area in the supernatant. The removal efficiency was calculated according to Equation (2) and the MIP with the highest efficiency for the aflatoxins was selected as the best option for extraction and was used in subsequent studies.

(2) $$R\;\left(\%\right)=\frac{C_{0}-C_{e}}{C_{0\;}}\times 100$$

where *C*_o_ is the initial analyte concentration and *C*_e_ is the equilibrium analyte concentration (mg/L).

The adsorption capacity (*Q*) (mg/g) and total adsorption capacity were calculated using Equations (3) and (4), respectively.

(3) $$Q_{e}=C_{o}-C_{e}\times \frac{V}{m}$$

(4) $$Q_{e}=\sum \;Q_{\mathrm{AFs}}$$

where *v* is the volume of the solution (L) and *m* is the mass of the adsorbent (g). The IF and the IF_avg_ were then defined according to Equations (5) and (6), respectively.

(5) $$IF=Q_{MIP}/Q_{NIP}$$

(6) $$IF_{\mathrm{avg}}=\sum IF_{\mathrm{AFs}}/4$$

### 3.6 Characterization

The functional groups in the molecules and molecular structure of both MIP and NIP were identified using FTIR and the polymer morphologies were examined using FESEM. The polymer thermal stability was examined using TGA.

### 3.7 Adsorption Studies

#### 3.7.1 Effect of Initial Concentration

The effect of initial aflatoxin concentration on the adsorption capacity of the MIP and NIP was investigated by adding 4 mg of the selected MIP into five aflatoxin solutions (10 mL) of varying concentrations (1, 2.5, 5, 10, 15 mg/L). Adsorption was allowed to occur for 1 h over a rotary shaker. The amount of each aflatoxin adsorbed per unit mass of the MIP was calculated using Equation (3). The same procedure was carried out to calculate the adsorption capacity of the NIP. The adsorption capacity at each spiking concentration was utilized in the nonlinear Langmuir isotherm model (Equation 7) to evaluate the binding energy (equilibrium constant) and number of available binding sites (maximum binding capacity). In addition, the *R*_L_ values (equilibrium parameter) associated with the Langmuir isotherm characteristics were calculated using Equation (8).

(7) $$Q_{e}=\frac{Q_{\max}K_{L}C_{e}}{1+K_{L}C_{e}}$$

(8) $$R_{L}=1/1+K_{L}C_{o}$$

where *C*_o_ is the initial aflatoxin concentration, *C*_e_ is the aflatoxin concentration at equilibrium, *Q*_e_ is the amount of aflatoxin adsorbed per unit mass of adsorbent at equilibrium, *Q*_max_ is the maximum adsorption capacity of the MIP, and *K*_L_ is the Langmuir constant.

#### 3.7.2 Effect of Contact Time

The effect of contact time on the *Q*_e_ was investigated from 5 to 60 min. A mass of 4 mg of each polymer was evenly dispersed in 10 mL of aflatoxin solutions, which were spiked at 5 mg/L. Adsorption was allowed to occur for the different contact times in an orbital shaker. Thereafter, the mixtures were centrifuged, the supernatant was collected, dried, and reconstituted with 1 mL MeOH/H_2_O (50:50 v/v), then filtered and injected into LC–MS/MS. The data obtained was used in explaining the rate and kinetic mechanism by plotting the linearized intraparticle diffusion, pseudo‐first‐, and second‐order models using Equations (9)–(11), respectively. The model with the highest *R* ^2^ value was used to explain the adsorption mechanism.

(9) $$Q_{t}=K_{\mathrm{dif}}\sqrt{t}+C$$

(10) $$\log\left(Q_{e}-Q_{t}\right)=\log\left(Q_{e}\right)-K_{1}\left(t/2.303\right)$$

(11) $$t/Q_{t}=1/K_{2}{\left(Q_{e}\right)}^{2}+t/Q_{e}$$

where *Q*_t_ is the amount of aflatoxins adsorbed at any given time *t*, *K*_dif_ is the intraparticle equilibrium rate constant, *C* is the intercept, *Q*_e_ is the amount of aflatoxins adsorbed at equilibrium time, and *K*_1_ and *K*_2_ are the pseudo‐first‐order and pseudo‐second‐order equilibrium rate constants, respectively.

#### 3.7.3 Effect of pH

A mass of 4 mg of MIP was added into a series of 15 mL centrifuge tubes containing 10 mL of 5 mg/L aflatoxins solution (MeOH/MeCN, 50:50 v/v) at pH 3,4, 5, 6, 7, 8, 9, 10, and 11. The pH of the aflatoxin solution was adjusted by adding dilute HCl and NaOH. The prepared solutions were shaken for 15 min and then centrifuged. The supernatant was collected, dried over a nitrogen stream, and reconstituted with 1 mL MeOH/H_2_O (50:50 v/v), then filtered and injected into LC–MS/MS. The concentrations of aflatoxins after the adsorption were determined, and the adsorption efficiencies of the MIP were calculated using Equation (2).

#### 3.7.4 Adsorption Thermodynamics

Adsorption thermodynamics were studied at three temperatures, 298, 318, and 338 K. Thermodynamic parameters such as the changes in the Gibbs free energy (Δ*G*°), enthalpy (Δ*H*°), and entropy (Δ*S*°) related to the adsorption process were calculated using the following equations.

(12) $$\Delta G^{\circ }=\Delta H^{\circ }-T\Delta S^{\circ }$$

(13) $$\ln K=\frac{-\Delta H^{\circ }}{RT}+\frac{-\Delta S^{\circ }}{R}$$

where *R* is the ideal gas constant (8.314 J/mol K), *K* (g/L) is the distribution coefficient, and *T* is the temperature in Kelvin. Δ*H*° (−slope × *R*) and Δ*S*° (intercept × *R*) were determined from the Van't Hoff plot (ln*K* vs. 1/*T*).

### 3.8 Selectivity Study

To ascertain the selective recognition and adsorption of aflatoxins by the selected MIP, a MeOH/MeCN (50:50 v/v) solution containing 5 mg/L of each of aflatoxins and other commonly co‐occurring mycotoxins (FB_1_, OTA, and ZEA) was mixed with 4 mg of the MIP. Adsorption was allowed to occur in an orbital shaker for 15 min. The solution was centrifuged, filtered, reconstituted, and injected into LC–MS/MS to determine the concentration of unadsorbed compounds. The aflatoxins were adsorbed from the mixture in the presence of other mycotoxins. The impact of imprinting on selectivity was then estimated using the distribution coefficient, *K*_D_ (mg/g) Equation (14).

(14) $$K_{D}=\left(C_{i}-\;C_{f}\right)\times V/W$$

where *C*_i_ is the initial concentration, *C*_f_ is the final concentration, *V* is the volume (L), and *M* (g) is the weight of the adsorbent. In addition, in the presence of competitors, the selectivity coefficient (K) for the binding of aflatoxins was estimated using Equation (15). Furthermore, M9's relative selectivity coefficient was determined using Equation (16).

(15) $$K=\frac{K_{D}\left(\mathrm{target}\right)}{K_{D}\left(\mathrm{competitor}\right)}$$

(16) $$K^{\prime }=\frac{K_{\mathrm{MIP}}}{K_{\mathrm{NIP}}}$$

## 4 Conclusion

In conclusion, this study has demonstrated the impact of different dummy templates on the extraction efficiency of MIPs for aflatoxins. The findings indicate that the preparation of MIPs using the right dummy templates produces MIPs with high adsorption capacity for targeted compounds. A selective and efficient MIP was prepared for the adsorption of aflatoxins using DMC as a template. Extraction recoveries > 70% were achieved for aflatoxins in the presence of competing mycotoxins. MIPs prepared using dummy templates can be integrated into green analytical chemistry (GAC) methods for sustainable contaminant monitoring strategies. Future research should explore advanced fabrication techniques, such as surface imprinting or nanocomposite integration, to further enhance binding kinetics, reusability, and stability under real‐world conditions. In addition, future research can explore more dummy templates monomers for the economic feasibility of the molecular imprinting process. Ultimately, this work highlights the importance of rational MIP design in developing robust materials for mycotoxin mitigation in food and agricultural applications.

## Conflicts of Interest

The authors declare no conflict of interest.

## Supporting information




**Supporting File 1**: cbdv70344‐sup‐0001‐SupMat.pdf

## Data Availability

The data that support the findings of this study are available in the Supporting Information of this article.
