# Supplementary material for: Synthesis and Characterization of Dummy Template‐Based Molecularly Imprinted Polymers for Extraction of Aflatoxins in Food Samples
Source: Chem Biodivers. 2025 Aug 9;22(12):e01749. doi: 10.1002/cbdv.202501749 (PMC12716015; doi:10.1002/cbdv.202501749)
Supplement: Supplementary file 1 — Supporting File 1: cbdv70344‐sup‐0001‐SupMat.pdf [file CBDV-22-e01749-s001.pdf]

Tables

Table S1: Adsorption performance of the studied polymers

Polymer	Template	Functional monomer	$(C_o\text{-}C_e) \pm$ SD (n =3) (mg/L)	Removal efficiency (R%)	Q (mg/g)	IF
M1	7-methoxy coumarin	MAA	1.60 ± 0.10	80.0	4.0	5.97
M2	1-hydroxy-2-naphthoic acid	MAA	1.13 ± 0.15	56.7	2.83	4.22
M3	7-hydroxy-3,4,8-trimethyl coumarin	MAA	1.37 ± 0.06	68.3	3.42	5.10
M4	7-methoxy coumarin	MAM	1.63 ± 0.15	81.7	4.08	5.44
M5	1-hydroxy-2-naphthoic acid	MAM	1.43 ± 0.06	71.7	3.58	4.77
M6	3-acetlycoumarin	MAA	1.03 ± 0.15	51.7	2.83	4.22
M7	7-hydroxy-3,4,8-trimethyl coumarin	MAM	1.40 ± 0.10	70	3.50	4.67
M8	3-acetlycoumarin	MAM	0.87 ± 0.06	43.3	2.17	2.89
M9	DMC	MAA	1.77 ± 0.06	88.3	4.42	6.60
M10	DMC	MAM	1.73 ± 0.06	86.5	4.33	5.77
N1	-	MAA	0.27 ± 0.12	13.3	0.67	-
N2	-	MAM	0.30 ± 0.10	15.0	0.75	-

Table S2: Pseudo-first-order model equations of straight lines and constants for adsorption of aflatoxins by M9 and NIP

Adsorbent	Aflatoxin	Equation of line	R^2	Q_e (mg/g) {$e^{intercept}$}	K_1 (min^{-1}) {-slope}
M9	B1	Y = -0.2276x – 0.49101	0.89457	0.612	0.02276
	B2	Y = -0.00603x + 0.34378	0.91301	1.410	0.00603
	G1	Y = -0.00985 – 0.02124	0.82973	0.990	0.00985
	G2	Y = -0.02234 – 0.0437	0.78278	0.978	0.0223

NIP	B1	Y = -0.00729x – 0.31377	0.87693	0.731	0.00729
	B2	Y = -0.00659x – 0.29602	0.80645	0.744	0.00659
	G1	Y = -0.00541x – 0.379	0.85432	0.685	0.00541
	G2	Y = -0.00794x – 0.1961	0.8670	0.822	0.00794

Table S3: Intraparticle diffusion constants for adsorption of aflatoxins by M9

Aflatoxin	Equation of straight line	K_{diff} (mg/g/$min^{1/2}$) {slope}	C {Intercept}	R^2
B1	y = 0.0746x + 7.6239	0.0746	7.62	0.6632
B2	y = 0.2388x + 4.7609	0.2388	4.76	0.8770
G1	y = 0.157x + 6.5249	0.157	6.52	0.730
G2	y = 0.2871x + 5.7489	0.2871	5.75	0.5864

Figures

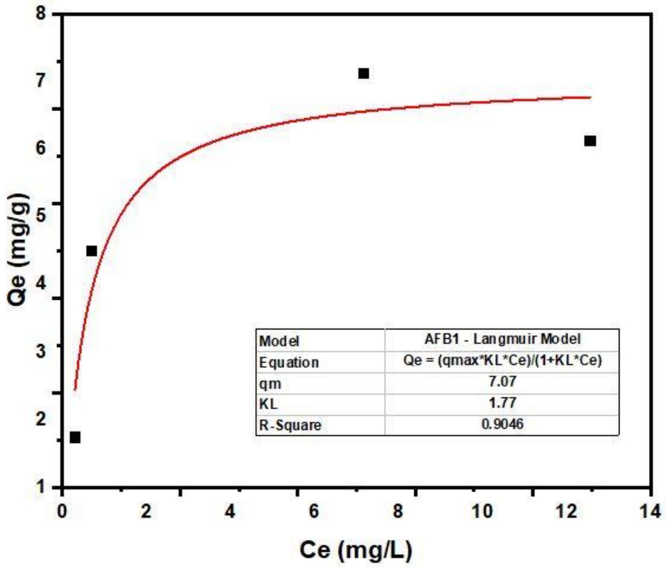

Figure S1: Langmuir isotherm for adsorption of AFB1 by M9

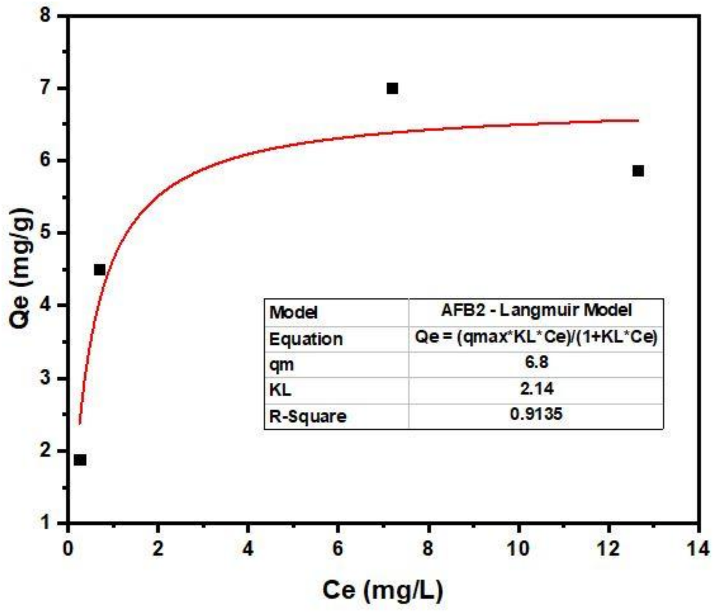

Figure S2: Langmuir isotherm for adsorption of AFB2 by M9

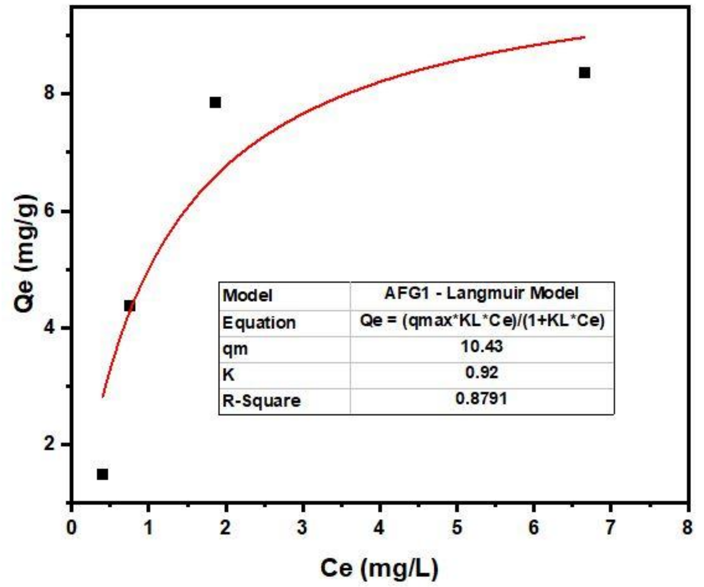

Figure S3:Langmuir isotherm for adsorption of AFG1 by M9

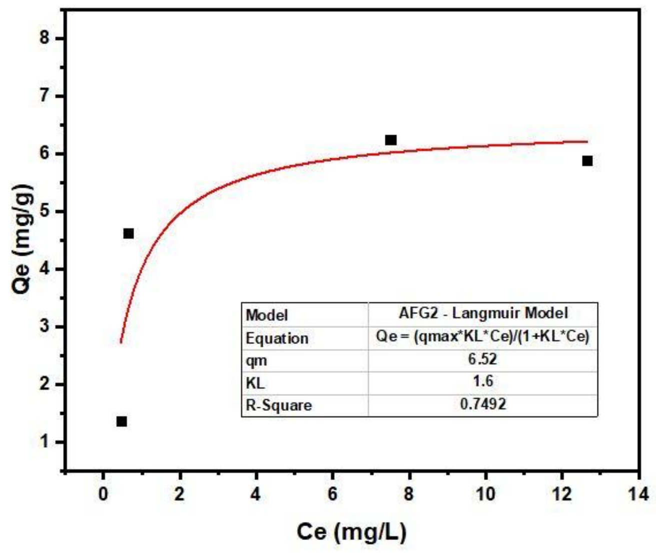

Figure S4: Langmuir isotherm for adsorption of AFG2 by M9

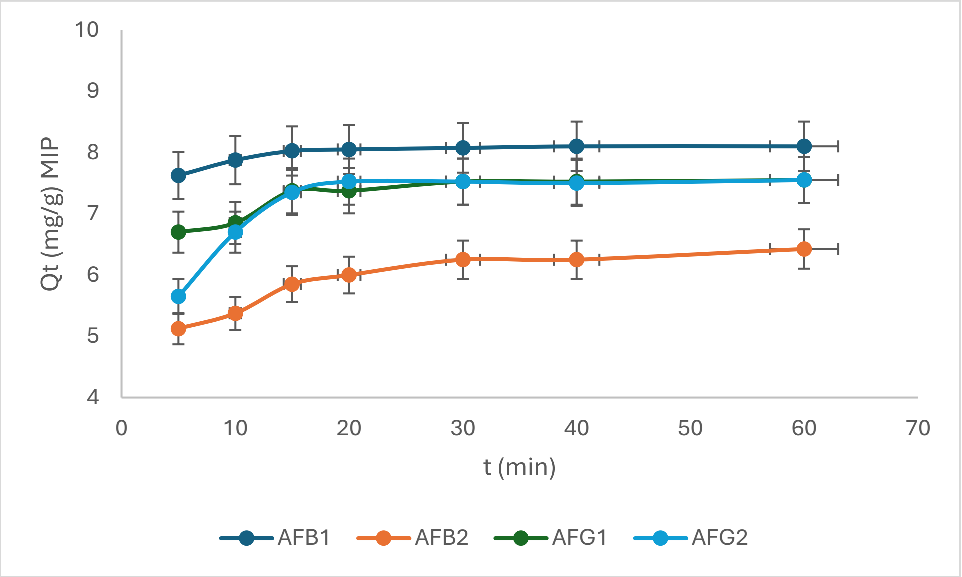

Figure S5: Batch binding analysis of M9 for aflatoxins (n=3, %RSD < 12%)

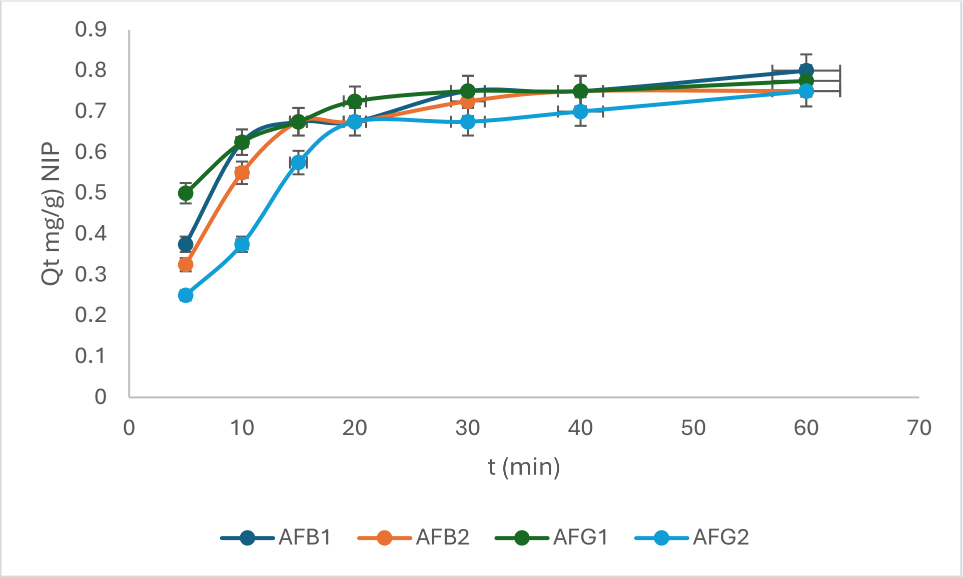

Figure S6: Batch binding analysis of NIP for aflatoxins (n=3, %RSD < 8%)

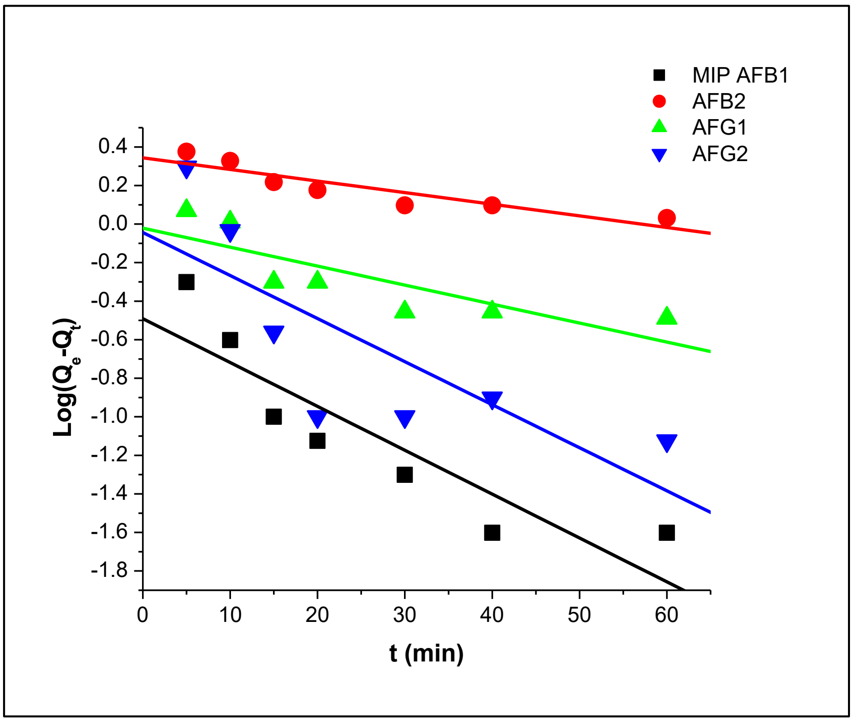

Figure S7: Pseudo first-order liner plots for M9

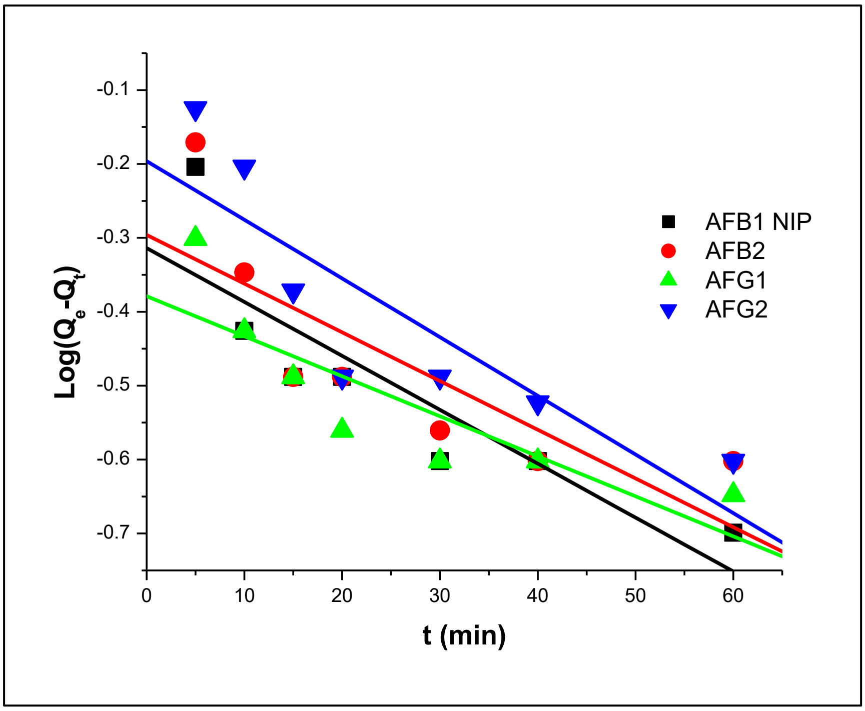

Figure S8: Pseudo first-order liner plots for NIP

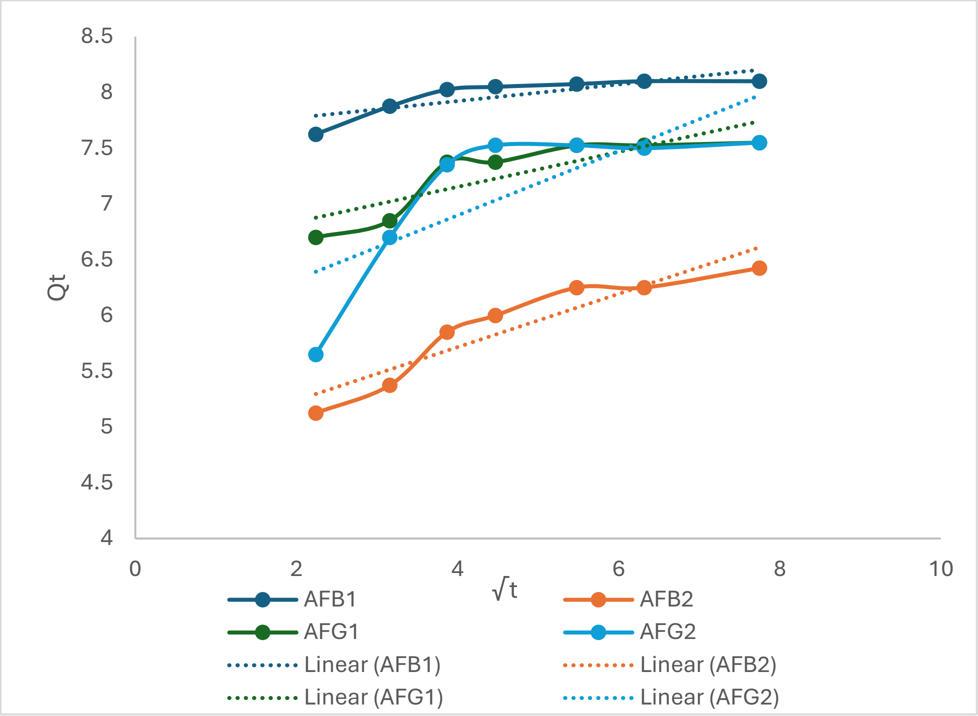

Figure S9: Intraparticle diffusion linear plots for adsorption of aflatoxins by M9

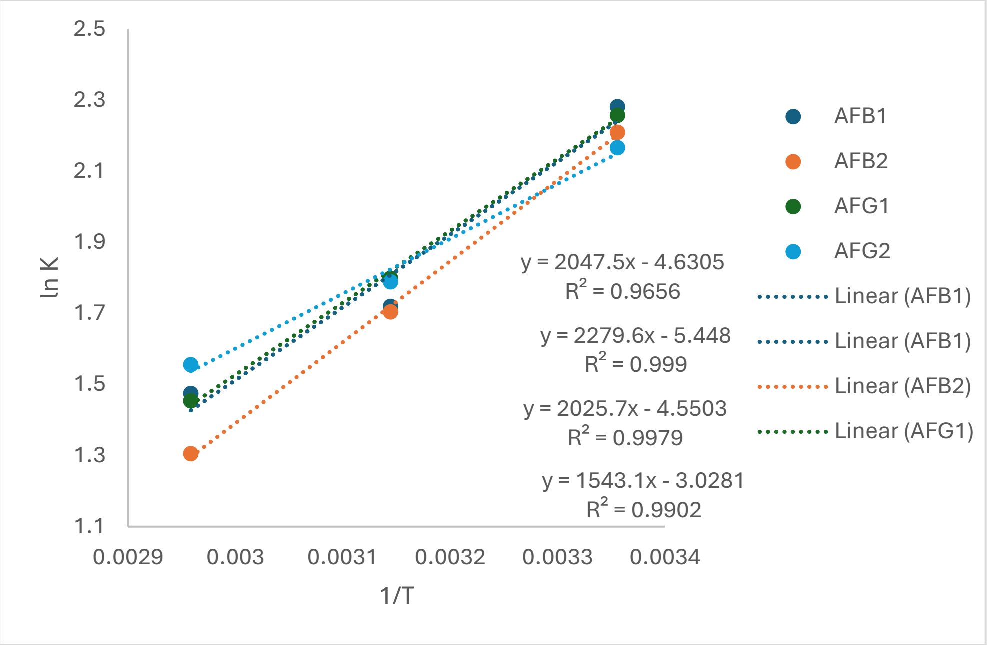

Figure S5: Van't Hoff plots for the adsorption of aflatoxins by M9
